# *Borrelia persica* infection in dogs and cats: clinical manifestations, clinicopathological findings and genetic characterization

**DOI:** 10.1186/s13071-016-1530-5

**Published:** 2016-05-10

**Authors:** Gad Baneth, Yaarit Nachum-Biala, Tamar Halperin, Yizhak Hershko, Gabriela Kleinerman, Yigal Anug, Ziad Abdeen, Eran Lavy, Itamar Aroch, Reinhard K. Straubinger

**Affiliations:** Koret School of Veterinary Medicine, Hebrew University, Rehovot, Israel; Medical Laboratory, Medical Corps, IDF, Tel Aviv, Israel; Pathovet LTD, Yehosa Ben Hanania 81, Rehovot, 76391 Israel; Faculty of Medicine, Department of community Health, Al-Quds University, Jerusalem, Palestinian Authority; Bacteriology and Mycology, Institute for Infectious Diseases and Zoonoses, Ludwig-Maximilians-Universität München, Munich, Germany

**Keywords:** Relapsing fever, Borreliosis, *Borrelia persica*, Feline, Canine

## Abstract

**Background:**

Relapsing fever (RF) is an acute infectious disease caused by arthropod-borne spirochetes of the genus *Borrelia*. The disease is characterized by recurrent episodes of fever that concur with spirochetemia. The RF borrelioses include louse-borne RF caused by *Borrelia recurrentis* and tick-borne endemic RF transmitted by argasid soft ticks and caused by several *Borrelia* spp. such as *B. crocidurae*, *B. coriaceae*, *B. duttoni*, *B. hermsii*, *B. hispanica* and *B. persica*. Human infection with *B. persica* is transmitted by the soft tick *Ornithodoros tholozani* and has been reported from Iran, Israel, Egypt, India, and Central Asia.

**Methods:**

During 2003–2015, five cats and five dogs from northern, central and southern Israel were presented for veterinary care and detected with borrelia spirochetemia by blood smear microscopy. The causative infective agent in these animals was identified and characterized by PCR from blood and sequencing of parts of the flagellin (*flab*), *16S rRNA* and glycerophosphodiester phosphodiestrase (*GlpQ*) genes.

**Results:**

All animals were infected with *B. persica* genetically identical to the causative agent of human RF. Phylogenetic analysis indicated that DNA sequences from these pet carnivores clustered together with *B. persica* genotypes I and II from humans and *O. tholozani* ticks and distinctly from other RF *Borrelia* spp. The main clinical findings in cats included lethargy, anorexia, anemia in 5/5 cats and thrombocytopenia in 4/5. All dogs were lethargic and anorectic, 4/5 were febrile and anemic and 3/5 were thrombocytopenic. Three dogs were co-infected with *Babesia* spp. The animals were all treated with antibiotics and the survival rate of both dogs and cats was 80 %. The cat and dog that succumbed to disease died one day after the initiation of antibiotic treatment, while survival in the others was followed by the rapid disappearance of spirochetemia.

**Conclusions:**

This is the first report of disease due to *B. persica* infection in cats and the first case series in dogs. Infection was associated with anemia and thrombocytopenia. Fever was more frequently observed in dogs than cats. Domestic canines and felines suffer from clinical disease due to *B. persica* infection and may also serve as sentinels for human infection.

## Background

Relapsing fever (RF) is an acute infectious disease caused by arthropod-borne spirochetes of the genus *Borrelia*. The disease in humans is characterized by recurrent episodes of fever, which usually concur with spirochetemia and is considered from a historical perspective the first disease linked to a specific microbial causative infectious agent [1]. The RF borrelioses can be grouped into two forms: louse-borne epidemic RF caused by *B. recurrentis* and tick-borne endemic RF commonly transmitted by argasid soft ticks with the exception of *Borrelia miyamotoi, * transmitted by several species of *Ixodes* hard ticks [2, 3].

Human infection with *B. persica* also known as Persian RF has been reported from Iran, Israel, Egypt, Pakistan, and former USSR Asian republics including Uzbekistan [4–8].

*B. persica* is transmitted by the soft tick *Ornithodoros tholozoni* whose distribution includes the Middle East, Central Asia and Northern India [9]. *Ornithodoros tholozani* feeds on warm-blooded animals and commonly lives in caves, ruins, rock crevices and man-made shelters where livestock is housed [10]. Human RF borreliosis is a reportable disease in Israel and the annual incidence average in civilians declined from 0.35 cases per 100,000 inhabitants from 1975 to 1985 to 0.11 cases per 100,000 inhabitants from 1986 to 2003. However, the incidence among Israeli soldiers is considerably higher with an average of 6.4/100,000 persons [11]. Although human RF borreliosis due to *B. persica* in the Middle East is usually not associated with mortality, severe infections with the acute respiratory syndrome and fatal infections have been reported in Israel [12, 13]. Three different genotypes of *B. persica* have been described from humans and *O. tholozani* ticks based on DNA sequences of the flagellin (*flaB*) gene [9].

Disease in domestic animals due to RF borreliae was rarely described. In this context, borreliosis with two species of RF borreliae, *B. turicatae* and *B. hermsii*, has been reported to cause disease in dogs in the USA [14–17]. *Borrelia persica* infection has recently been reported in a young spirochetemic puppy from Iran [18]. However, to our best knowledge no descriptions of disease caused by RF borreliae have been reported in domestic felines except for the isolation of *B. persica* from an Israeli cat, which is one of the cases described in this study [19].

## Methods

### Naturally-infected animals

Spirochetemia was detected during microscopic examination of blood smears stained by Romanowsky staining solutions from animals whose blood was submitted for a complete blood count (CBC) to veterinary diagnostic laboratories in Israel or performed in-house by veterinarians during the years 2003 to 2015. Blood anticoagulated in EDTA from these animals was submitted for molecular identification and genotyping to the Koret School of Veterinary Medicine at the Hebrew University.

### PCR and genetic analysis

DNA was extracted from 200 μl of EDTA-anticoagulated blood samples from spirochetemic animals using the Illustra blood genomicPrep Mini Spin Kit (GE Health care, Buckinghamshire, UK), following the manufacturer’s instructions. PCRs were performed with primers targeting three different genes of RF borreliae (Table 1). An approximately 515 bp fragment of the *Borrelia* 16S ribosomal RNA (*16S rRNA*) gene was amplified using primers rec4 and rec9 as previously described [20]. An approximately 346 bp fragment of the *flab* gene was amplified from extracted DNA samples as previously reported for other *Borrelia* species using primers Bfpbu and Bfpcr [21], and an approximately 212 bp fragment of the glycerophosphodiester phosphodiesterase (*GlpQ*) gene, which is specific for RF borreliae, was amplified using primers 128f and 340r [22]. DNA from a borrelia-negative dog and from a human case of *B. persica* infection were used as negative and positive controls, respectively. A non-template negative control (NTC) was also included in each PCR run. Positive DNA amplicons were purified (EXO-Sap, New England Biolabs Inc., Ipswich, USA) and sequenced in the Center for Genomic Analyses at the Hebrew University (Jerusalem, Israel) using the BigDye Terminator cycle from Applied Biosystems ABI3700 DNA Analyzer. The ABI Data Collection and Sequence Analysis software (ABI, Carlsbad, USA) was used for analysis. DNA sequences were compared for similarities to other sequences in GenBank using the BLAST program hosted by NCBI, National Institutes of Health, USA (http://www.ncbi.nlm.nih.gov) and new DNA sequences from *Borrelia-*infected dogs and cats were deposited in GenBank.

**Table 1:** Target genes and primers used for PCR to detect and characterize *Borrelia persica* in blood samples from cats and dogs

Target gene	Primers	Primer sequence (5’ to 3’)	Reference
*16S rRNA*	REC4	ATGCTAGAAACT GCATGA	[20]
	REC9	TCGTCTGAGTCCCCATCT	
*flab*	Bfpbu	GCT GAA GAG CTTGGAATGCAACC	[21]
	Bfpcr	TGATCAGTTATCATTCTAATAGCA	
*GlpQ*	128f	CAGAACATACCT TAGAAGCTCAAGC	[22]
	340r	GTGATTTGATTTCTGCTAATGTG	

PCRs for *Ehrlichia canis* and for *Babesia* spp. were performed on DNA from the dog blood samples using primers 16S-F and 16S-R and the Piroplasmid-F and Piroplsamid-R primers, respectively, as previously described [23, 24]. PCR for hemotrophic *Mycoplasma* species was performed using primers HBT-F and HBT-R as previously described [25]. Serology for feline leukemia virus (FeLV) and for feline immunodeficiency virus (FIV) was performed using a commercial assay (SNAP FIV/FeLV Combo Test, IDEXX Laboratories, Westbrook, Maine, USA).

### Phylogenetic analysis

A phylogenetic analysis, which included DNA sequences from this study, was carried out to compare these sequences to other *Borrelia* spp. that had previously been deposited in GenBank. Sequences were analyzed using the MEGA version 6.1 (http://www.megasoftware.net/) and phylogenetic trees were constructed by the maximum likelihood algorithms using the Tamura-3-Parameter model [26]. Bootstrap replicates were performed to estimate the node reliability, and values were obtained from 1000 randomly selected samples of the aligned sequence data.

## Results

The demographic and clinical findings from the ten spirochetemic cats and dogs are presented in detail in Tables 2 and 3. The five cats were from cities (Jerusalem and Arad) and from rural villages (Kfar Adumim, Matzuba and Kfar Oranim) in southern, central and northern Israel and all had outdoor access. The five dogs were all from small towns and villages (Yavne’el, Meitar, Hashmonaim, Karmei Yosef and Amatzia) located also in southern, central and northern Israel.

**Table 2:** Demographic and clinical characteristics of cats infected with *Borrelia persica* included in the study

Cat number (sample ID)	1 (6812)	2 (42798)	3 (9727)	4 (213813)	5 (8738)
Location	Kfar Adumim	Jerusalem	Arad	Matzuba	Kfar Oranim
Year of diagnosis	2003	2003	2010	2012	2015
Outdoor access	+	+	+	+	+; stray
Sex; age in years	F; 2	M; 1	Male; ND	F; 7.5	F; 1
Fever	–	–	–	-	39.8 °C
Lethargy	+	+	+	+	ND
Anorexia	+	+	+	+	ND
Pale mucous membranes	–	–	+	+	+
Icterus	–	–	+	+	-
Anemia	+	+	+	+	+
Hematocrit L/L	0.208	0.116	0.113	0.109	0.270
MCV fL	40.5	42.1	31.2	39.3	ND
MCHC g/L	336	288	389	390	ND
Leukocytosis; WBC number × 10^9^/L	–; 11.6	–; 5.86	+; 33.88	+; 21.33	-; Est WNL
Thrombocytopenia; PLT number × 10^9^/L	+; 35	+; 119	–; 227	+; 71	+; Est low
Co-infection: PCR for hemotrophic *Mycoplasma* spp. Serology FeLV; FIV	–	–	–	-	-
	(–; –)	(–; –)	ND	(-; +)	(-; -)
Antibiotic treatment	amoxicillin/clavulanic acid	amoxicillin/clavulanic acid	long acting tetracycline and amoxicillin/clavulanic acid	doxycycline	doxycycline
Survival and response to treatment	survived; spirochetemia not evident one day after treatment initiation	died one day after treatment initiation	survived	survived; clinical improvement reported 12 h after initial doxycycline; clinical recovery in 4 days	survived; recovered clinically and no spirocehtemia evident on follow-up 21 days after treatment initiation

*ND* not determined, *Est* estimated from blood smear, *WNL* within normal limits; Hematocrit, reference range 0.277–0.468 L/L; *MCV* mean cell volume, range 41–53 fL; *MCHC* mean corpuscular hemoglobin concentration, range 270–330 g/L; *WBC* white blood cells, range 6.3–19.6 × 10^9^/l; PLT: platelets, range 156–626 × 10^9^/l

**Table 3:** Demographic and clinical characteristics of dogs infected with *Borrelia persica* included in the study

Dog number (sample ID)	1 (61887)	2 (8726)	3 (4663)	4 (9835)	5 (8692)
Location	Yavne' el	Meitar	Hashmonaim	Karmei Yosef	Amatzia
Year of diagnosis	2003	2012	2012	2013	2015
Sex; age in years	M; 1.5	F; 3	M; 4	F; 12.5	M; 2.5
Breed	Siberian Husky	Mixed breed	Labrador	Mixed breed	German Shepherd
Fever	40.8 ° C	41.2 °C	39.5 °C	–; (38.9 ° C)	39.4 °C
Lethargy	+	+	+	+	+
Anorexia	+	+	+	+	+
Pale mucous membranes	–	+	+	+	-
Icterus	–	–	+	–	-
Anemia	+	+	+	+	-
Hematocrit L/L	0.271	0.340	0.189	0.270	0.375
MCV fL	75.3	77.7	66.7	69.2	67.5
MCHC g/l	305	305	337	353	381
Leukocytosis	–	–	–	–	-
WBC number × 10^9^/l	10.69	11.80	12.80	9.13	7.70
Thrombocytopenia	–	+	–	+	+
PLT number × 10^9^/l	168	41	171	86	39
Co-infection:					
PCR for *E. canis*	–	–	–	–	-
PCR for *Babesia* sp.	–	+	+	+	-
Antibiotic treatment	ciprofloxacin for 2 days and then doxycycline	doxycycline	long acting tetracycline and amoxicillin/clavulanic acid	amoxicillin initially, continued with doxycycline	doxycycline
Survival and response to treatment	survived; no spirochetemia at recheck 7 days after treatment initiation	survived; no spirochetemia at recheck 11 days after treatment initiation; treated also with imidocarb dipropionate for babesiosis	died one day after treatment initiation	survived; improved a day after initial treatment with amoxicillin; treated also with imidocarb dipropionate for babesiosis	survived; recovered clinically at recheck 4 days after treatment initiation

Hematocrit, reference range 0.371–0.57 L/L; *MCV* mean cell volume, range 58.8–71.2 fL; *MCHC* mean corpuscular hemoglobin concentration, range 310–362 g/l; *WBC* white blood cells, range 5.2–13.9 × 10^9^/L; PLT: platelets, range 150–400 × 10^9^/l

The five spirochetemic cats included three females and two males with an age range of one to 7.5 years. Only one of the cats had fever (> 39.0 °C). All four cats with owners presented with lethargy and anorexia. Three of the five cats had pale mucous membranes and two were icteric. Blood for CBC was available from four cats (#1–4) and only hematocrit and blood smear evaluation were recorded from cat # 5. All cats were anemic (hematocrit < 0.277 L/L) with microcytic anemia in three cats. Thrombocytopenia was evident in 4/5 cats (platelets < 156 × 10^9^/l) and confirmed by blood smear evaluation, while leukocytosis (> 19.6 × 10^9^/l) was evident in 2/5. All cats were negative for hemotrophic *Mycoplasma* species by PCR. Four of the cats (#1, 2, 4 and 5) were tested for infection with FeLV and FIV and cat #4 was found positive for FIV. Additional blood was not available to test cat # 3 for infection with these retroviruses. All cats received antibiotic treatment; three were treated with amoxicillin/clavulanic acid, two with doxycycline and one with a combination of amoxicillin/clavulanic acid and long acting injectable tetracycline. The cats that survived the infection were treated for variable durations ranging from one to four weeks. One cat (#2) died one day after treatment initiation, whereas the remaining four cats survived and recovered. Cat # 1 did not have evident spirochetemia when evaluated by blood smear one day after treatment initiation; the owners of cat # 4 reported a clinical improvement twelve hours after initial antibiotic administration and return to normal activity within four days; and cat #5 also recovered clinically rapidly and no spirochetemia was detected on a follow-up 21 days after treatment initiation.

The five spirochetemic dogs included three males and two females with an age range of 1.5 to 12.5 years. Four had fever (> 39.0 °C) and the other dog had a borderline fever of 38.9 °C. All dogs presented lethargy and anorexia. Four of the five dogs had pale mucous membranes and one was icteric. Four dogs were anemic (hematocrit < 0.371 L/L) with macrocytic anemia in two. Thrombocytopenia (< 150 ×10^9^/l) was evident in three animals. All dogs were negative for *E. canis* infection tested by PCR and three (#2,3 and 4) were positive for *Babesia* spp. by PCR and blood smear evaluation. All dogs received antibiotic treatment: two were treated with doxycycline alone, one with ciprofloxacin for two days and then switched to doxycycline, another one received a combination of long acting tetracycline and amoxicillin/clavulanic acid, and one received amoxicillin initially and was later switched to doxycycline. Two dogs with babesiosis (#2 and 4) were treated also with imidocarb dipropionate injections. The dogs that survived the infection were treated with doxycycline for 21 days. One dog (#3) co-infected with *Babesia* sp. died one day after treatment initiation, whereas the four other dogs survived and fully recovered. Dogs #1 and #2 did not have evident spirochetemia in their blood smears at recheck seven and eleven days after treatment initiation, respectively. In dogs #4 and #5, clinical improvement was noted one and four days, respectively, after initial antibiotic administration.

**Fig. 1:**
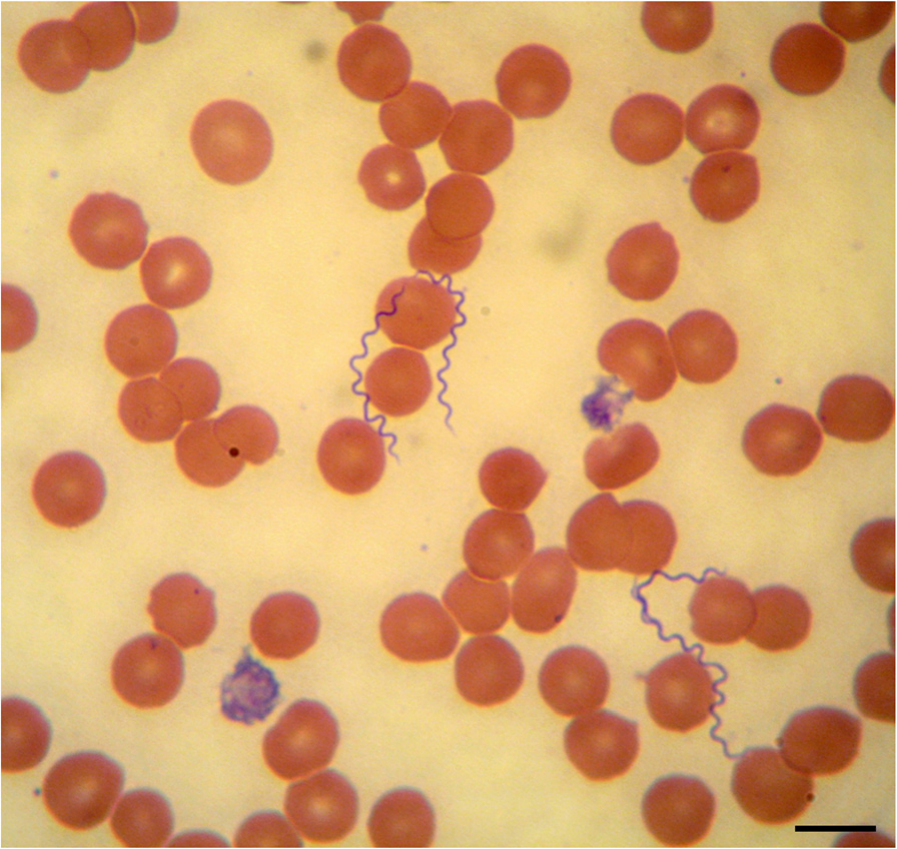
Spirochetemia with *Borrelia persica* in a blood smear from dog no. 4. Romanowsky stain. *Scale-bar*: 10 μM

**Fig. 2:**
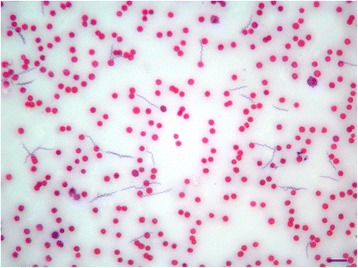
Prominent *Borrelia persica* spirochetemia in cat no. 2. Romanowsky stain. *Scale-bar*: 20 microns

**Fig. 3:**
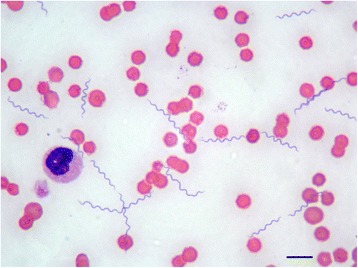
Higher magnification of spirochetemia with *Borrelia persica* in cat no. 2. Romanowsky stain. *Scale-bar*: 10 μM

**Fig. 4:**
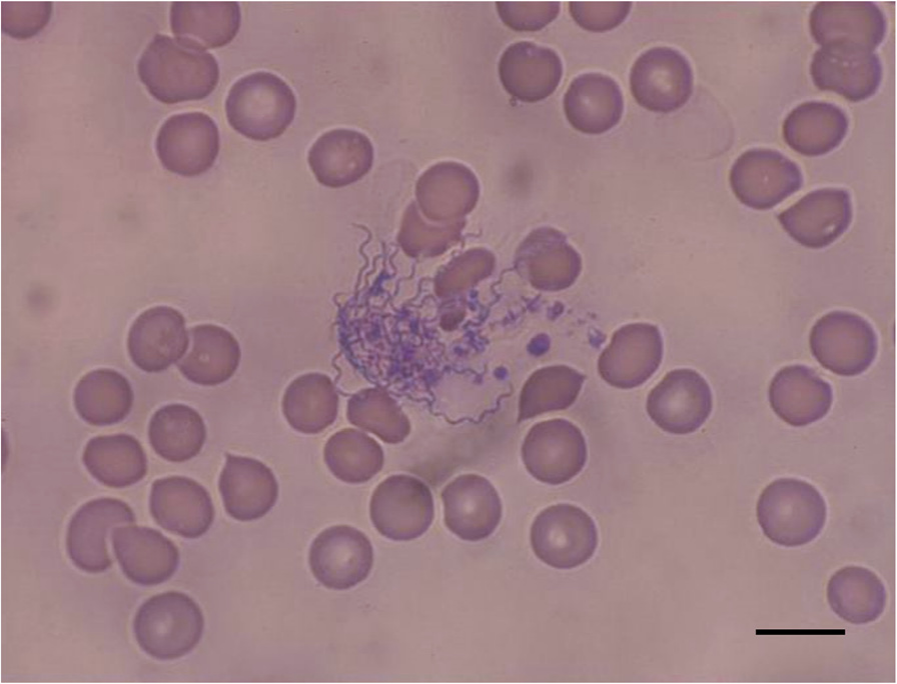
*Borrelia persica* spirochetes aggregating with platelets in a blood smear from dog no. 1. Romanowsky stain. *Scale-bar*: 10 μM

**Fig. 5:**
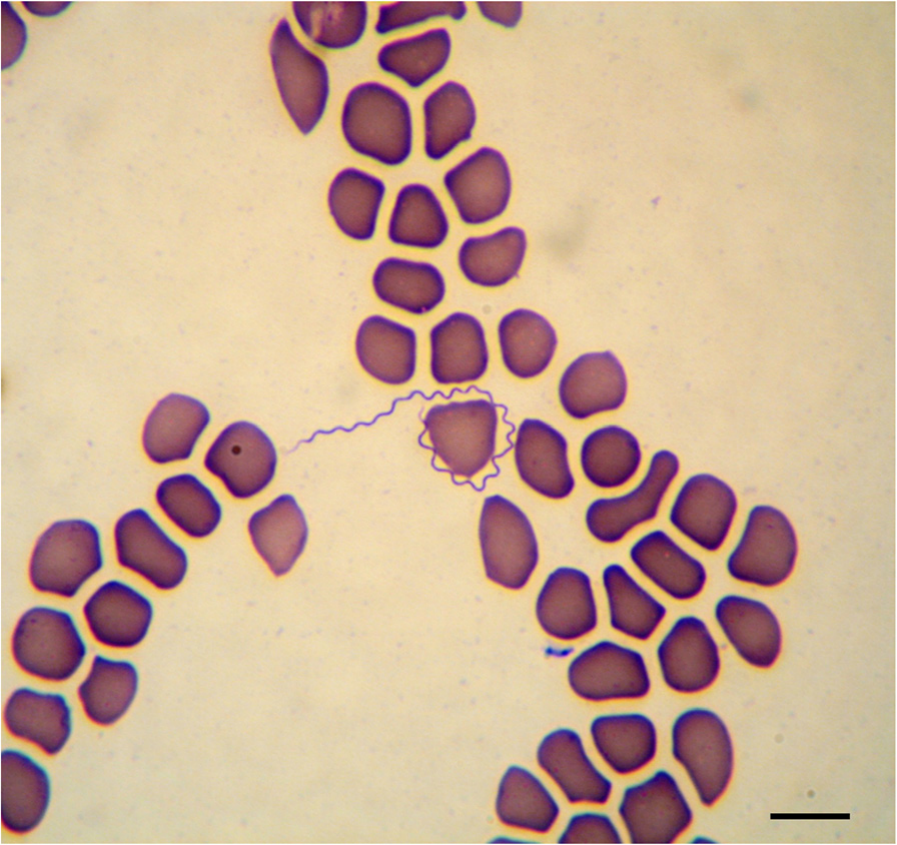
*Borrelia* persica spirochete encircling a canine erythrocyte in blood smear from dog no. 2. Romanowsky stain. *Scale-bar*: 10 μM

Numerous spirochetes were noted in blood smears from all dogs and cats with 4 to 10 spirochetes per microscopic field at 500× magnification (Fig. 1). Cat #2 which died in the course of infection had an overwhelmingly high spirochetemia (Figs. 2 and 3) and blood obtained from this cat was used for culture of *B. persica* as previously described [19]. Spirochetes seen in the blood smears occasionally formed aggregates with platelets (Fig. 4) or encircled erythrocytes (Fig. 5).

PCR of blood from all cats and dogs was positive for *Borrelia* sp. by all three PCR protocols amplifying fragments of the *16S rRNA*, *flaB* and *GlpQ* genes, except for cat # 2. This animal was not tested with the *GlpQ* PCR, because no further DNA was available for the test (Table 4). DNA sequences amplified from all of the animals with the three PCR protocols were 99–100 % identical to *B. persica* sequences already deposited in GenBank and *B. persica* was constantly the first match in all BLAST searches. Twenty-nine DNA sequences from this study were deposited in GenBank and assigned accession numbers (Table 4).

**Table 4:** GenBank accession number of *Borrelia persica* from cats and dogs included in the study

Animal number	16S rRNA	flagellin (*flab*)	glycerophosphodiester phosphodiesterase (*GlpQ*)
Cat 1 (6812)	DQ207601	KT895509	KT895516
Cat 2 (42798)	DQ207603	DQ211356	ND
Cat 3 (9727)	KT895504	KT895511	KT895518
Cat 4 (213813)	KT895503	KT895510	KT895517
Cat 5 (8738)	KU565880	KU565881	KU932019
Dog 1 (61887)	DQ207600	DQ207604	KU932020
Dog 2 (8726)	KT895505	KT895512	KT895519
Dog 3 (4663)	KT895506	KT895513	KT895520
Dog 4 (9835)	KT895507	KT895514	KT895521
Dog 5 (8692)	KT895508	KT895515	KT895522

*ND* not done

The *Babesia* PCR products amplified by the Piroplasmid PCR from dogs #2, 3 and 4 yielded sequences that differed from other known *Babesia* spp. in GenBank and therefore were termed *Babesia* sp. in this study and await further characterization.

**Fig. 6:**
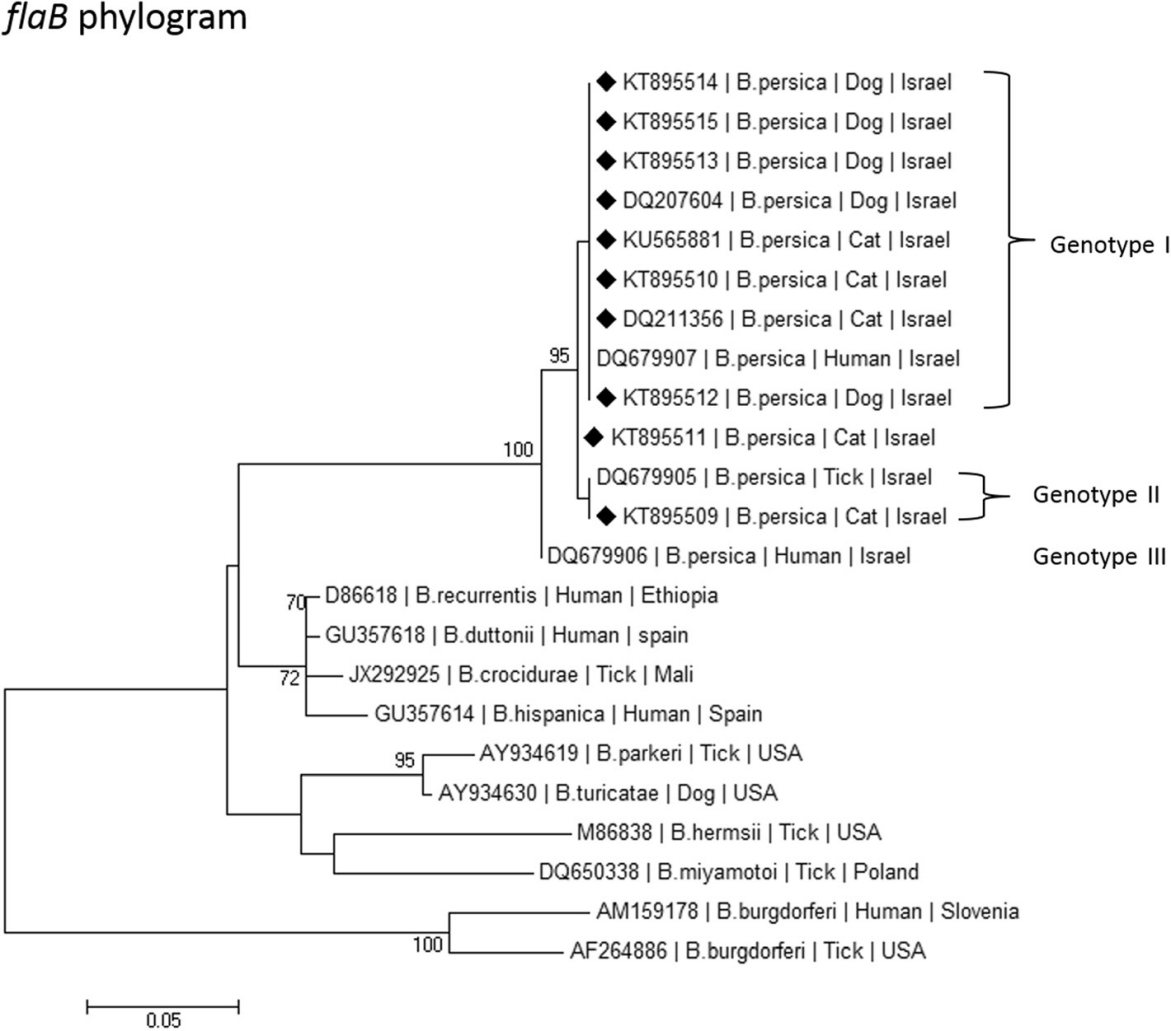
A maximum likelihood phylogram comparing 267 bp DNA sequences of the *flaB* gene from the cats and dogs included in the study to sequences from other *B. persica* GenBank accessions and from other *Borrelia* spp. New sequences derived from this study are marked with black diamond squares. Note the division into *B. persica* genotypes marked in Roman numerals. The GenBank accession numbers, species of infected host and country of origin are included for each sequence. The Tamura-3-Parameter model was used in the construction of this phylogram and bootstrap values higher than 70 % are indicated

Phylogenetic analysis of the *flaB* gene sequences (Fig. 6) indicated that the sequences from the *B. persica* infected dogs and cats clustered together with each other and with sequences of other *B. persica* organisms recovered from humans and *O. tholozani* ticks. Most of the cat and dog sequences clustered together with a *B. persica* genotype 1 sequence (DQ679907) from a human individual, whereas one cat sequence clustered together with a *B. persica* genotype 2 from a tick (DQ6795509), while none of our sequences clustered closely with *B. persica* genotype 3 from a human (DQ679906). All *B. persica* sequences clustered separately from other Old World RF *Borrelia* spp. including *B. duttonii*, *B. crociduare*, *B. hispanica* and *B. recurrentis*. RF species from the American continent including *B. parkeri*, *B. turicatae* and *B. hermsii* also clustered separately and together with *B. miyamotoi* which was described from the American continent and also from Asia and Europe.

**Fig. 7:**
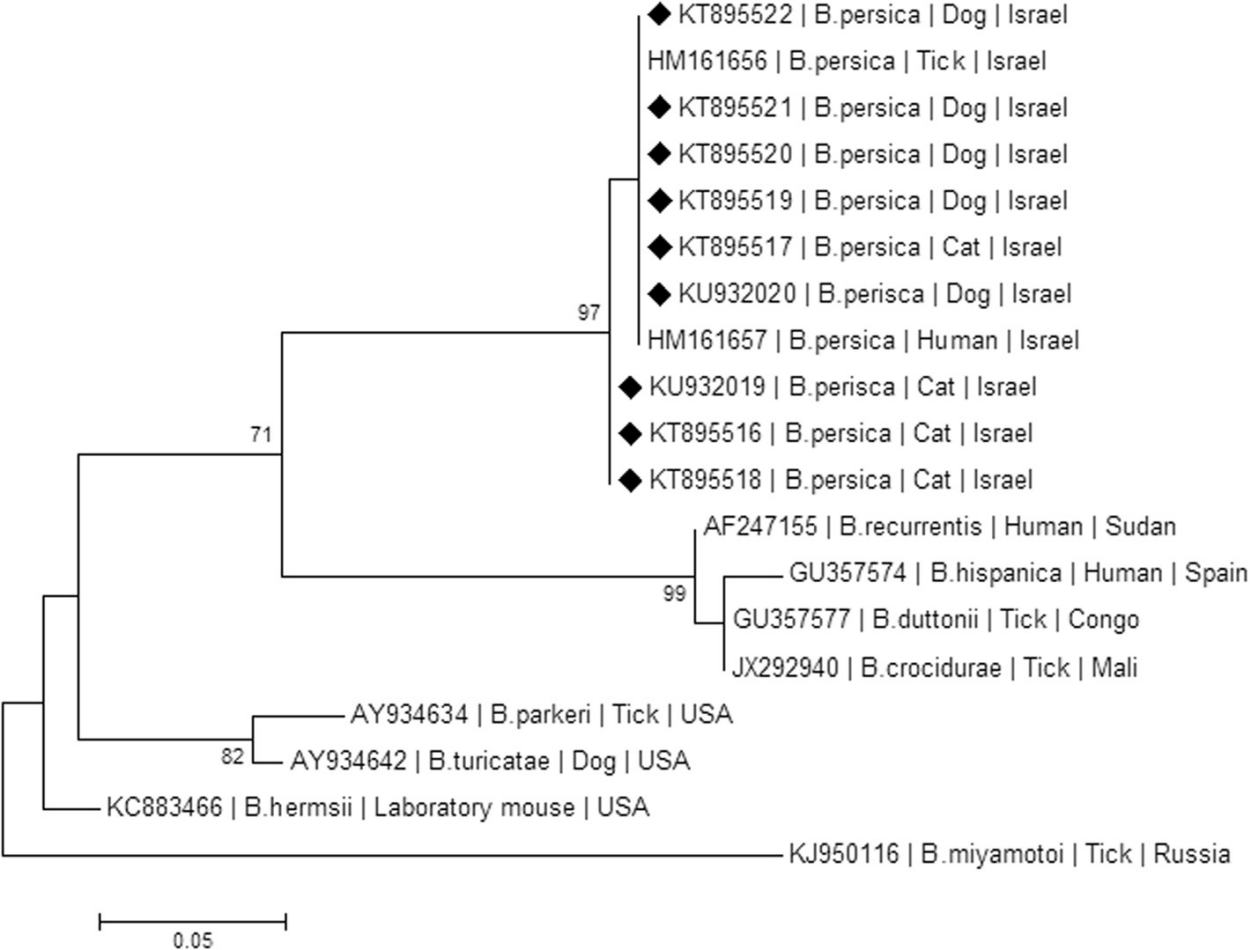
A maximum likelihood phylogram comparing 133 bp DNA sequences of the *GlpQ* gene from the cats and dogs included in the study to sequences from other *Borrelia persica* GenBank accessions and from other *Borrelia* spp. New sequences derived from this study are marked with black diamond squares. The GenBank accession numbers, species of infected host and country of origin are included for each sequence. The Kimura-2-Parameter model was used in the construction of this phylogram and bootstrap values higher than 70 % are indicated

Phylogenetic analysis of *GlpQ* sequences (Fig. 7) also indicated that the sequences from the *B. persica*-infected dogs and cats clustered together with each other and with other *B. persica* sequences from a human and an *O. tholozani* tick. As in the *flaB* phylogram, *B. persica GlpQ* sequences clustered separately from Old World RF *Borrelia* spp. including *B. hispanica*, *B. duttonii*, *B. crocidurae* and *B. recurrentis*. The Old World species including *B. persica* also clustered separately from American RF *Borrelia* spp. namely *B. hermsii*, *B. parkeri* and *B. turicatae*.

## Discussion

This study describes clinical disease associated with *B. persica* infection in domestic cats and dogs. The infected animals had profound spirochetemia, were lethargic, anorectic and suffered from anemia and frequent thrombocytopenia. Infected dogs were febrile or borderline febrile, whereas fever was noted in only one infected cat. Treated dogs and cats mostly recovered rapidly with antibiotic treatment, and spirochetemia apparently cleared as found in follow up blood tests. Nevertheless, the fact that two of the infected animals (20 %) had died indicates that this disease is not benign and is potentially fatal in pets. The Jarisch–Herxheimer reaction with fever, sweating, anorexia and occasional death upon initiation of antibiotic treatment for RF borreliosis and bacterial decay, has been associated with the increase of circulating levels of tumor necrosis factor α (TNF-α), interleukin-6, and interleukin-8 in humans [27]. Death due to a similar reaction may have occurred also in the dog and cat in this study that died soon after the beginning of antibiotic treatment. No evidence of cyclic spirochetemia with relapsing episodes of fever, as shown in infected people, was reported for the infected dogs and cats. Yet, this may be due to the difficulty of getting a thorough history on these pets, the possibility that the infected animals were treated and not given the opportunity to develop recurrent fever episodes, which could be recorded by their veterinarians, or simply due to lack of close monitoring.

The only previous report of a dog with infection caused by *B. persica* was described in a 2-week old puppy from Teheran, Iran, presenting anorexia, pale mucous membranes, diarrhea, vomiting and anemia [18]. Another species, *B. hispanica* has been shown to experimentally infect a dog by rat bite [28]. Two other species of RF borreliae have been reported to infect dogs in North America. Dogs with *B. turicatae* infection reported from Texas and Florida [14–16] were febrile, lethargic, anorectic, anemic and thrombocytopenic. A single case of canine infection with *B. hermsii* was reported from Washington State, USA. This dog presented with lethargy, anorexia, fever, anemia, leukopenia and thrombocytopenia [17]. These reports from dogs infected with *B. turicatae*, *B. hermsii* and the *B. persica*-infected puppy from Iran indicate that different RF *Borrelia* spp. are able to infect dogs with similar clinical manifestations including lethargy, anorexia, fever, anemia and thrombocytopenia.

Disease caused by *B. persica* does not seem to be frequent in Israeli cats and dogs as the cases included in this series, although probably not the only cases of this disease in Israel during the period of case collection, had been recorded over a 12-year period. Furthermore, the lack of apparent geographical clustering of cases and the wide distribution of disease locations from the southern part of the country to its north, suggests that infection is sporadic.

The common treatment for human *B. persica* RF is with doxycycline [29, 30] although treatment with amoxicillin has also been recommended [9]. Doxycycline is also recommended as the major drug for post-exposure prevention [31, 32]. Although almost all the dogs and cats in this study, which recovered from disease were treated with doxycycline or another tetracycline, cat #1 also recovered and was apparently non-spirochetemic one day after treatment initiation with amoxicillin and clavulanic acid, whereas dog #4 was treated initially with amoxicillin, reported to improve one day later, and only then was continued with doxycycline. This suggests that several antibiotics may be effective against *B. persica* infection in cats and dogs. Nevertheless, due to the apparent efficacy of doxycycline evident in this study and based on the recommendation for human treatment and prophylaxis with this drug [29, 31], it would be sensible to recommend doxycycline as a first line antibiotic for canine and feline *B. persica* infection.

Co-infection with babesiosis potentially contributed to the severity and clinical manifestations of *B. persica* infection in the three co-infected dogs. Canine babesiosis is also associated with fever, anemia and thrombocytopenia [33]. Nevertheless, the two dogs with no detectable babesiosis were also febrile, thrombocytopenic (dog # 5) or borderline thrombocytopenic (dog # 1) and anemic (dog #1) or borderline anemic (dog #5). Thus, babesiosis by itself cannot account for all the clinical findings in the dogs with *B. persica* infection. Furthermore, no cats were positive for hemotrophic mycoplasmas and only one of the four cats tested for FeLV and FIV was positive for FIV (#4). Therefore, no clear association of feline *B. persica* infection with hemortophic *Mycoplasma* or immune-suppressive viral infections such as FeLV and FIV is apparent.

The genetic analysis of *B. persica* from cats and dogs based on three different genes strongly suggests that the pathogen from these animals is identical to the cause of RF in humans in Israel and other countries. The *B. persica* organisms detected in the animals belonged to two of the three known *B. persica* genotypes described from humans in Israel [5, 9]. Since the same infectious agent exists in humans and domestic animals, the infection and disease might be considered a zoonosis. Nevertheless, the question of transmission and life-cycle of *B. persica* is complex as transovarial transmission of this infection from the adult female tick via the eggs to its offspring has been reported [34], and thus this infection might not need an animal reservoir. Consequently, the roles of animals or humans in the life-cycle of this pathogen may only be the supply of a blood meal for the host tick. Despite this, this bacterium is adapted to growth in medium containing human serum [19] and also to propagation in animals as shown experimentally in laboratory mice [35]. Therefore, *B. persica* may easily infect animals and hence vertebrates may play an important role in its life-cycle as reservoir hosts.

The tick *O. tholozani* is typically found in caves, ruins and archeological sites in Israel where human infection with *B. persica* has often been reported to be acquired, and the disease is frequently referred to as cave fever [9, 11]. Since *O. tholozani* ticks feed rapidly and do not commonly attach to their host for more than 20–30 min [9], ticks are usually not found on human patients and are also not reported on animal hosts in Israel. Therefore, it would be unlikely to find cats and dogs infested with *O. tholozani*. Furthermore, in perspective of the nature of the tick’s habitat, it seems more probable that wildlife animals would serve as reservoirs of this infection, as cats and dogs are not expected to reach remote cave locations, which are far from their typical environment. Such potential wildlife reservoirs may nevertheless be related to dogs or cats, e.g. wild canids such as jackals, foxes or wolves, or wild feline species.

Although we have documented severe disease with profound *B. persica* spirochetemia in dogs and cats, chronic persistent sub-clinical low level spirochetemia undetected by conventional blood smear microscopy may exist in animals, which could serve as reservoirs for tick infection. Such a possibility should be evaluated by PCR surveys. Such surveys of animal infection with *B. persica* could be of public health importance as animals may serve as sentinels for human infection.

## Conclusions

This study describes severe clinical potentially fatal disease associated with *B. persica* infection, a causative agent of human RF, in domestic cats and dogs. Infection was associated with lethargy, anorexia, anemia and thrombocytopenia in both species while fever was more frequent in infected dogs than in cats. This infection therefore endangers both humans and domestic carnivores, and in the context of One Health, animal infection may serve as a sentinel for human infection.

### Animal ethics statement

This study was carried out in accordance with the Hebrew University ethic regulations for experimentation in animals. The study involved exclusive use of residual blood samples taken as a part of the animals diagnostic procedure by attending veterinarians.

## Abbreviations

*16S rRNA*: 16S ribosomal RNA gene
CBC: complete blood count
FeLV: feline leukemia virus
FIV: feline immunodeficiency virus
*flab*: flagellin gene
*GlpQ*: glycerophosphodiester phosphodiestrase gene
RF: relapsing fever
